# The Tuberculosis Congress

**Published:** 1920-05-22

**Authors:** 


THE PUBLIC WELL-BEING—(*continued*).

### THE TUBERCULOSIS CONGRESS.

The two points chiefly emphasised at the Tuberculosis Congress held last week at Leeds under the auspices of the Tuberculosis Society of Great Britain and Ireland were (1) the serious effect which the disease exercises upon the child-life of the country, and (2), the apparent hopelessness of adequately combating its ravages in the vast slums of our industrial areas. Dr. C. W. Vining, assistant physician-in-charge of the Children's department at the Leeds General Infirmary, told the Congress that tuberculosis is one of the most frequent causes of mortality among children under the age of ten. The greatest time of danger is during the second and third years. From that time onward the disease rapidly diminishes as a cause of death. Dr. Vining, like most physicians and social workers, is appalled by the amount of breakdown that occurs in the second year of life, leading to a grave weakening of the child's chief line of defence against the tubercle bacillus; and as the main defects are the want of breast feeding during the first year of life and a diet lacking in fat and protein, with excess of carbohydrate during the second year of life, he is convinced that no considerable improvement can take place until the mother is compelled to place herself under a competent authority during the child's first year. Every child born should be registered under such an authority, as well as merely noted by the Medical Officer of Health; and the contact between the parents and the authorities should be maintained until the child passes into the hands of the school authority.

A Difficult Ideal.

But though such supervision and a wide and persistent dissemination of knowledge may do much to mitigate the evil, the deadly infection of tubercle will never be stamped out and the "preventable" disease will never become a "prevented" disease as long as our great towns abound in slum areas of the worst description and in that odious abomination—back-to-back houses—of which there are nearly 80,000 in Leeds alone. Dr. Halliday Sutherland, of the Marylebone Tuberculosis Dispensary, pointed out that there is four times more tuberculosis in the poorer parts of a city than in the better-off parts, which means that tuberculosis is, to a very large extent, a disease of poverty. We agree with his ideal—but almost despair of reaching it in an overcrowded country which depends for its existence upon purely industrial activities—that "everything should be done to discourage the growth of cities and to encourage the growth of semi-agricultural and semi-industrial communities, as in France."

Some controversy arose, as was to be expected, over the subject of milk as a means of conveying infection. Mr. S. W. Daw, clinical lecturer in surgery at Leeds University, declared that statistics proved that more than half the milking cows are infected with tuberculosis, and that 15 per cent. carry infection in their milk. On the other hand, Dr. H. Ellis (Middlesbrough) boldly contended that to increase the power of resistance to the disease is a vastly more important factor than the risk of infection. Experience at Sir Henry Gauvain's hospital at Alton and elsewhere has convinced him that invariably the tissue is stronger than the germ, and thus to his mind the whole question is one of raising the power of resistance. Unsterilised milk is admittedly superior to the sterilised variety, and the

**THE PUBLIC WELL-BEING**—(*continued*).

point which he made was that the withdrawal of vitamines strikes a vital blow at this resisting-power. The problem may be solved by a new electrical sterilisation process, which could retain the vitamines. But even so, Dr. Ellis would remain an enthusiastic advocate of non-sterilisation. In spite of the disagreement among the experts, the careful mother will probably be wise in continuing to boil milk, even when she has reason to believe that the source is perfectly pure, and to replace the essential vitamines by alternative foods.

